# Selective Heck Arylation of Cyclohexene with Homogeneous and Heterogeneous Palladium Catalysts 

**DOI:** 10.3390/molecules15042166

**Published:** 2010-03-25

**Authors:** Ewa Mieczyńska, Anna M. Trzeciak

**Affiliations:** Faculty of Chemistry, University of Wrocław, 14 F. Joliot-Curie, 50-383 Wrocław, Poland; E-Mail: gosia@wchuwr.pl (E.M.)

**Keywords:** palladium, Heck coupling, heterogenized catalysts, cyclohexene

## Abstract

Palladium catalysts containing Pd(II) supported on Al_2_O_3_ and alumina-based mixed oxides, Al_2_O_3_-ZrO_2_, Al_2_O_3_-CeO_2_, and Al_2_O_3_-Fe_2_O_3_, are very effective in the Heck coupling of iodobenzene with cyclohexene in DMF solution. The best results, up to 81% of monoarylated products with a selectivity to 4-phenylcyclohexene (**3**) close to 90% were obtained with KOH as a base. The catalytic activity of palladium supported on alumina-based oxides was compared with that of homogeneous precursors, such as Pd(OAc)_2_ and PdCl_2_(PhCN)_2_, used in [Bu_4_N]Br as the reaction medium. Under such conditions homogeneous systems were more selective and produced up to 60% of monoarylated products with a selectivity to **3** close to 60%.

## 1. Introduction

The Heck coupling, a C–C bond formation process also described as olefin arylation, is one of the most important palladium-catalyzed reactions [1,2,3,4,5,6]. Arylated olefins, the final products of the Heck reaction, have very broad application in the synthesis of pharmaceuticals, agrochemicals, and natural products. Therefore, very intensive research has been addressed to the development of new, simple and efficient catalytic systems for the Heck coupling.

Typical palladium catalysts for the Heck reaction contain phosphines; however, recently, phosphine-free catalytic systems, economical and environmentally friendly, have been receiving increasing attention [7,8,9,10,11,12,13,14,15,16]. 

It seems most likely that in phosphine-free systems a key role is played by Pd(0) nanoparticles formed *in situ* from Pd(II) precursors [4,9,14,15]. However, in the absence of any stabilizing agents such catalysts can be deactivated as a result of the formation of inactive “palladium black”. Tetraalkylammonium salts or inorganic supports are known as good stabilizers of palladium, often used in catalytic systems to prevent their deactivation [8,11,17,18,19,20]. 

There are also many examples of application in Heck coupling of palladium supported on inorganic oxides of Al, Ti, Zn, Zr, Mg [21,22,23,24,25,26], on silica [27,28] as well on zeolites [29,30]. In most cases reactions of aryl halides with acrylates, acrylonitrile, styrene or terminal alkenes were studied [31]. Mechanistic studies performed in these systems led to the conclusion that the main catalytic role is played by soluble palladium species formed as a result of leaching from the support and re-adsorbed in the end of the catalytic process [32].

The Heck reaction of cyclic olefins, like cyclohexene, cyclopentene, or 2,3-dihydrofuran, present an attractive pathway to create molecules containing stereogenic centers [20,33,34,35,36,37,38,39,40]. The difficulty of these reactions has to do with their regioselectivity, because a mixture of different products, mono- and diarylated olefins, is always formed. Studies of the Heck coupling of cyclohexene with 4-bromoacetophenone have revealed the high catalytic activity of soluble palladium species, such as palladacycles or Pd(OAc)_2_/PPh_3_, which produce 89% and 79% of monoarylated olefins. Surprisingly only very few reports deal with application of heterogeneous systems in Heck coupling of cycloalkenes. For example, different heterogeneous palladium catalysts, i.e. Pd/C, Pd/SiO_2,_ Pd/MgO, Pd/Al_2_O_3_, have been used in Heck coupling of 4-bromoacetopheneone with cyclohexene and cyclo-pentene [33]. In contrast to homogeneous systems, heterogeneous catalysts were active not only in the Heck coupling but also in dehalogenation of the substrate leading to the formation of acetophenone as the predominant product [33].

In this paper, we present the results of our studies on the Heck coupling of iodobenzene with cyclohexene performed in homogeneous and heterogeneous phosphine-free systems. We chose palladium supported on alumina-based oxides as heterogeneous catalysts which shown already good catalytic performance in other cross-coupling reactions [41,42]. 

## 2. Results and Discussion

### 2.1. Homogeneous catalysts

**Figure 1:**
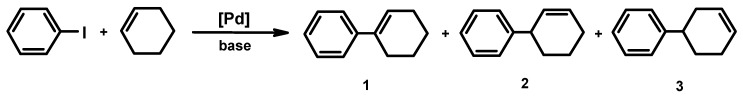
The Heck reaction of cyclohexene with iodobenzene.

**Figure 2:**
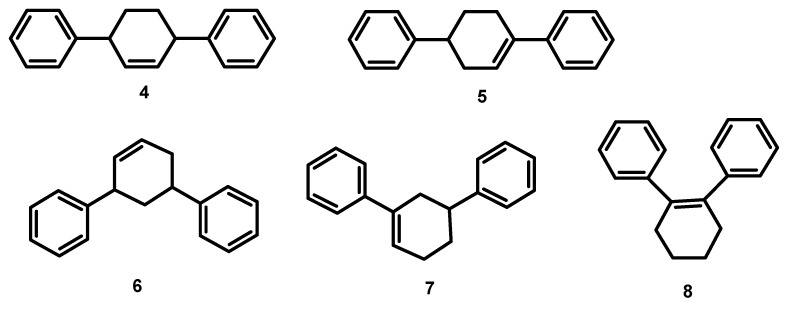
Diarylated products formed in the Heck reaction of cyclohexene with iodobenzene.

The Heck reaction of cyclohexene with iodobenzene is presented on Figure 1. According to literature data, three monoarylated products, **1, 2,** and **3**, may be formed, with 4-phenylcyclohexene (**3**) being the most thermodynamically stable [33]. Besides monoarylated products, diarylated ones can also be formed, and Figure 2 shows their five possible isomers.

Two palladium complexes, Pd(OAc)_2_ and PdCl_2_(PhCN)_2_, were tested in the Heck reaction under selected reaction conditions (140 ºC, 4 h) in DMF as solvent and produced less than 15% of products. The most plausible explanation for such low activity is a fast deactivation of the catalyst caused by its reduction to palladium black. The successful Heck coupling procedures of cycloalkenes with aryl halides reported up to now consisted in application of phosphorus ligands, preventing formation of inactive black palladium [33,34,35,36,37,38,39,40]. Therefore, in subsequent experiments molten [Bu_4_N]Br was used as the reaction medium. A positive effect of tetrabutylammonium salt was demonstrated earlier in other palladium catalyzed systems [11,20,43] and it was expected that also in the studied systems [Bu_4_N]Br would stabilize reduced palladium species (*i.e*. nanoparticles) formed *in situ*.

**Figure 3:**
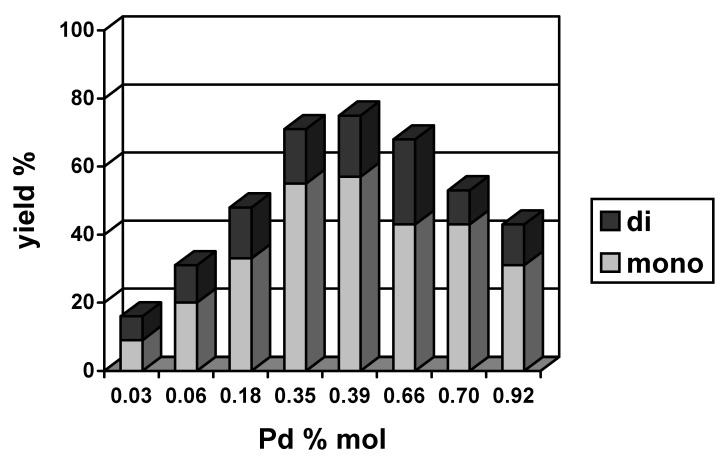
The yield of mono- and diarylated cyclohexenes obtained in the Heck reaction catalyzed by Pd(OAc)_2_ in [Bu_4_N]Br (140 ºC, 4 h, NaHCO_3_).

**Figure 4:**
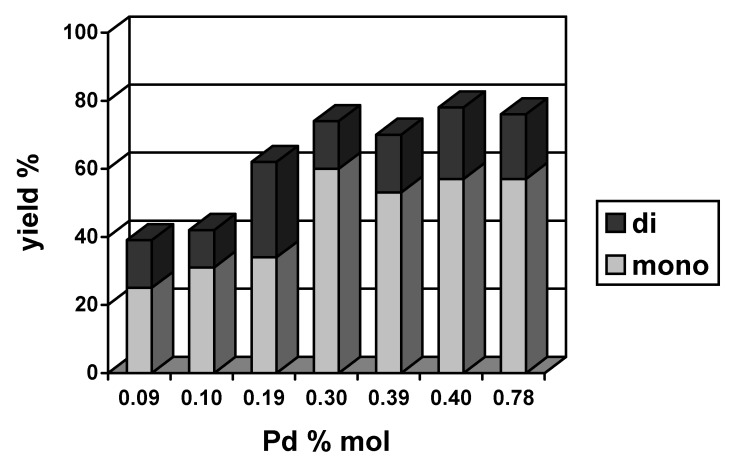
The yield of mono- and diarylated cyclohexenes obtained in the Heck reaction catalyzed by PdCl_2_(PhCN)_2_ in [Bu_4_N]Br (140 ºC, 4 h, NaHCO_3_).

Figure 3 and Figure 4 present the correlations between the total yield of the Heck reaction and the amount of palladium for both of the systems studied. Characteristically, in a case of Pd(OAc)_2_ the dependence was non-linear: the yield increased with an increase in the amount of palladium and then decreased. This type of concentration dependence had been observed earlier in other Heck reactions and interpreted as an indication of the participation of Pd(0) nanoparticles in the reaction course [44]. The formation of Pd(0) nanoparticles can be considered in the systems studied in agreement with XPS results that shown, that when PdCl_2_(PhCN)_2_ reacted with NaHCO_3_ in the presence of [Bu_4_N]Br *ca.* 50% of palladium was reduced to Pd(0) [22]. Most probably, Pd(0) nanoparticles stabilized by [Bu_4_N]Br were formed, similar to these described for Pd-chitosan nanocomposite [45]. In the presence of [Bu_4_N]Br and PhI palladium nanoparticles are dissolved forming soluble complexes of Pd(II), also catalytically active. A special role in the catalytic process is played by soluble complexes containing aryl ligands, e.g. [Bu_4_N]_2_[Pd(Ar)X_3_], key intermediates in the catalytic cycle [8].

From the dependences shown in Figure 3 and Figure 4, it is possible to estimate the optimal range of the palladium amount in both systems. For Pd(OAc)_2_, the highest yields of coupling products were obtained when the amount of palladium was 0.35–0.39% mol. For PdCl_2_(PhCN)_2_, the range of applicability was wider, 0.30–0.78% mol, and an effect of the amount of palladium was less significant. 

The yield of monoarylated products was similar for both catalysts, the maximum being 60% and the average amount of diarylated products being *ca.* 18%. In Table 1 there are collected results illustrating an effect of base on the reaction yield and selectivity. In the presence of NaOAc the yield of diarylated products increased to *ca.* 30%, whereas amount of monoarylated cyclohexenes reminded close to 50%. When KOH was used as a base 60–67% of monoarylated products were formed. As expected, product **3** was the main one in the fraction of monoarylated cyclohexenes and the highest selectivity to **3** was noted for KOH as a base. 

**Table 1:** Results of the Heck coupling of iodobenzene with cyclohexene catalyzed by Pd(OAc)_2_ and PdCl_2_(PhCN)_2_ in [Bu_4_N]Br.

Catalyst	Base	PhI conversion (%)	Yield 1+2+3 (%)	Selectivity 1:2:3	Yield 4+5+6+7+8 (%)	Biphenyl (%)
Pd(OAc)_2_	NaHCO_3_	74	55	16:20:64	16	4
	NaOAc	90	53	17:21:62	27	9
	KOH	100	67	13:3:84	23	10
PdCl_2_(PhCN)_2_	NaHCO_3_	81	53	19:19:62	17	11
	NaOAc	88	53	18:28:54	30	15
	KOH	100	60	15:5:80	20	20

*Reaction conditions*: 2.68 × 10^-3^ mol of PhI, 8.77 × 10^-3^ mol of cyclohexene, 0.53% mol Pd, 2.5 × 10^-3^ mol of NaHCO_3_, 5 cm^3^ of DMF or 2.3 × 10^-3^ mol of [Bu_4_N]Br, 140 ºC, 4 h.

### 2.2. Heterogeneous catalysts

For further studies, we selected heterogenized palladium catalysts obtained by impregnation of PdCl_2_ on alumina-based oxides. Alumina and three different alumina-based oxides containing 10% of ZrO_2_, or Fe_2_O_3_ or 2% of CeO_2_, were used as supports. The presence of the second metal oxide modifies the physicochemical properties of alumina, which can influence the catalytic activity. For example, an effect of CeO_2_ on acid-base properties of alumina was observed [46] and an increase of the hydrothermal stability of mixed Al_2_O_3_-ZrO_2_ oxides was noted [47,48]. It was also expected, that application of four different alumina-based oxides as supports for palladium catalysts would allow to formulate some more general conclusions about the catalytic activity of heterogeneous systems in the arylation of cyclohexene. 

First, the effect of the solvent was studied for one selected catalyst, Pd/Al_2_O_3 _+ CeO_2_, at different palladium concentrations (Table 2). When the amount of palladium increased from 0.26% mol to 0.35% mol, both the conversion of iodobenzene and the yield of monoarylated products **1–3** increased in DMF as solvent. The maximum yield of products **1–3** was 46% with selectivity to **3** of 74%. In these reactions, the yield of diarylated olefins and biphenyl remained at a similar level, 4–9%. When instead of DMF molten [Bu_4_N]Br was used as the reaction medium, the conversion increased to 79–91%, but selectivity decreased remarkably. The monoarylated products were obtained with a yield of *ca.* 40%, whereas the amount of diarylated olefins increased to *ca.* 30% and that of biphenyl to 16%. It is interesting to note that selectivity in the fraction of monoarylated products also changed and product **2** became the main one, whereas **3** predominated in reactions performed in DMF. 

**Figure 5:**
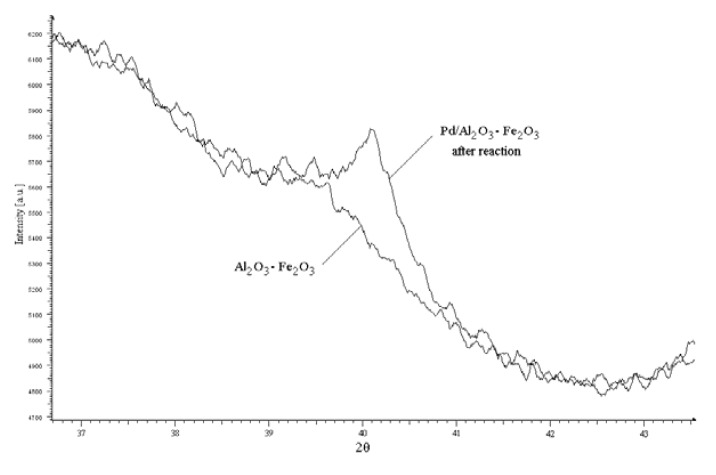
XRD pattern of Al_2_O_3_-Fe_2_O_3_ support and Pd/Al_2_O_3_-Fe_2_O_3_ after Heck reaction.

**Figure 6:**
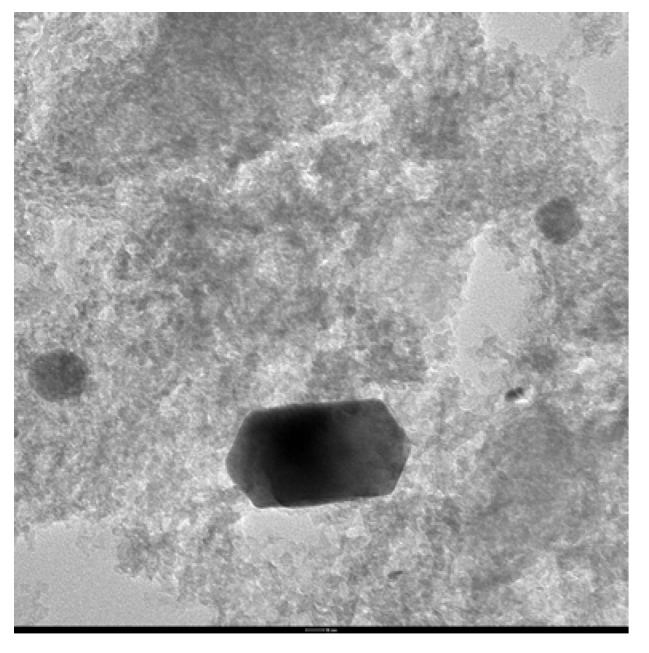
TEM of the Pd/Al_2_O_3_-Fe_2_O_3_ after Heck reaction.

In agreement with earlier results an intensive leaching of palladium from the support can be proposed for the catalytic system in [Bu_4_N]Br, leading to formation of anionic species of the type [Bu_4_N]_2_[PdX_4_], similar to these present in a homogeneous system. However, the different composition of final products of Heck coupling formed with homogeneous and heterogeneous palladium precursors in tetrabutylammonium salt can indicate that supported palladium is also involved in the reaction course. XRD and TEM analyses of one selected catalyst Pd/Al_2_O_3_-Fe_2_O_3_ performed after Heck reaction confirmed the presence of Pd(0) nanoparticles of diameter *ca.* 17 nm (XRD) or 15–50 nm (TEM) (Figure 5 and Figure 6).

The effect of PPh_3_ on the Heck coupling was also studied for comparison; however, it can be described as an inhibiting one, lowering the yield of monoarylated products (Table 1). Such an inhibiting effect can be explained to some extent by the reducing properties of PPh_3_. However PPh_3_ can also occupy active sites on the surface of the catalyst making it less accessible for coordination of reactants. 

**Table 2:** Results of the Heck coupling of iodobenzene with cyclohexene catalyzed by Pd/Al_2_O_3_+CeO_2_ in DMF and in [Bu_4_N]Br.

Pd% mol	Solvent	[PPh_3_]:[Pd]	PhI conversion (%)	Yield 1+2+3 (%)	Selectivity 1:2:3	Yield 4+5+6+7+8 (%)	Biphenyl (%)
0.26	DMF		39	31	16:13:71	4	5
0.26	[Bu_4_N]Br		79	32	16:47:38	31	15
0.35	DMF		43	33	15:12:73	5	6
0.35	[Bu_4_N]Br		91	45	13:49:38	30	16
0.53	DMF		64	42	14:10:76	10	12
0.53	[Bu_4_N]Br		85	40	13:57:39	29	16
0.35	DMF	5	27	17	18:12:70	4	5
0.35	DMF	10	32	22	18:5:77	4	5

*Reaction conditions*: 2.68 × 10^-3^ mol of PhI, 8.77 × 10^-3^ mol of cyclohexene, 2.5 × 10^-3^ mol of NaHCO_3_, 5 cm^3^ of DMF or 2.3 × 10^-3^ mol of [Bu_4_N]Br, 140 ºC, 4 h.

Further studies were continued in DMF as a solvent offering a higher selectivity in Heck coupling. Four palladium catalysts supported on different mixed-oxides used without any additives, produced up to 50% of monoarylated products. To check whether any improvement of selectivity was possible, different bases were tested for the catalyst Pd/Al_2_O_3 _(Table 3). With Cs_2_CO_3_ and K_2_CO_3_, an increase in biphenyl yield was noted, even up to 29%. Much better results were found when KOH or NaOH was used, KOH being superior to NaOH if the total yield of monoarylated cyclohexenes and selectivity to product **3 **are taken into account. A similar improvement in yield and selectivity was also noted for other palladium catalysts supported on Al_2_O_3_+ ZrO_2_ and Al_2_O_3_+ Fe_2_O_3_, which gave 78% and 81% of monoarylated products when KOH was used as a base. Moreover, selectivity to **3** exceeded 80%

**Table 3:** Results of the Heck coupling of iodobenzene with cyclohexene catalyzed by Pd supported on alumina-based oxides in DMF.

Catalyst	Base	PhI conversion (%)	Yield 1+2+3 (%)	Selectivity 1:2:3	Yield 4+5+6+7+8 (%)	Biphenyl (%)
Pd/Al_2_O_3 _+ ZrO_2_	NaHCO_3_	66	41	17:3:80	9	14
	KOH	89	78	13:3:84	4	7
Pd/Al_2_O_3 _+ CeO_2_	NaHCO_3_	64	42	14:10:76	10	12
	KOH	90	67	14:4:82	18	15
Pd/Al_2_O_3 _+ Fe_2_O_3_	NaHCO_3_	63	47	15:6:79	7	8
	KOH	98	81	11:1:88	12	5
Pd/Al_2_O_3 _	NaHCO_3_	58	37	16:8:76	7	14
	NaOAc	57	43	16:7:77	7	8
	K_2_CO_3_	86	48	15:12:73	9	29
	NaOH	90	53	17:4:79	22	15
	KOH	88	65	12:0:88	12	11

*Reaction conditions*: 2.68 × 10^-3^ mol of PhI, 8.77 × 10^-3^ mol of cyclohexene, 0.53% mol Pd, 2.5 × 10^-3^ mol of NaHCO_3_, 5 cm^3^ of DMF, 140 ºC, 4 h.

## 3. Experimental

### 3.1. Preparation of alumina-based supports

Alumina-based oxides were prepared by the sol-gel technique [41,42]. The proportion of ZrO_2_, Fe_2_O_3_ and CeO_2_ to Al_2_O_3_ was equal 10:100, 10:100 and 2:100. Specific surface area estimated according to BET method was *ca.* 200 m^2^/g. 

### 3.2. Preparation of supported Pd(II) catalysts

First, the support (0.8 g) was impregnated, with stirring, in an aqueous acidic solution (8 cm^3^, C_HCl_ = 0.09 mol/dm^3^) of PdCl_2_, containing 40 mg of Pd. After 72 h, the solution was decanted, and the Pd(II)-containing catalyst was washed three times with water and dried. The palladium content was estimated by the ICP method after mineralization of the weighted sample with *aqua regia.*The amount of palladium was from 1.3 to 1.9 wt % in the final catalysts.

### 3.3. Heck reaction procedure

The Heck reactions of iodobenzene with cyclohexene were carried out in a 50 cm^3^ Schlenk tube with magnetic stirring. The reagents: the catalyst, PdCl_2_(PhCN)_2_ (2.5 × 10^-6^–2.09 × 10^-5 ^mol) or Pd(OAc)_2_ (8.46 × 10^-7^–2.01 × 10^-5^ mol) or Pd supported on alumina-based oxide (7.0 × 10^-6^, 9.4 × 10^-6^ or 1.41 × 10^-5^ mol), PhI (0.3 cm^3^, 2.68 × 10^−3^ mol), cyclohexene (0.5 cm^3^, 8.77 × 10^−3^ mol), base (NaHCO_3_, NaOAc, K_2_CO_3_, KOH, NaOH, 2.5x10^-3 ^mol), and the solvent, [Bu_4_N]Br (0.75 g, 2.3 × 10^−3^ mol) or DMF (5 cm^3^), were introduced to the Schlenk tube under an N_2_ atmosphere. The tube was closed, and the reaction was carried out at 140 ºC for 4 h. Afterwards, organic products were separated by extraction with diethyl ether (twice with 10 cm^3^) and analyzed by GC–MS (Hewlett Packard 8452A) with dodecane (0.1 cm^3^, 4.4x10^-4^ mol) as internal standard. For reactions performed in DMF, diethyl ether (15 cm^3^) was added, and a sample was quenched with water (10 cm^3^). Organic products were extracted from the water phase with diethyl ether (15 cm^3^), and a combined ether solution was analyzed by GC. Products **1, 2,** and **3** were identified by comparison of their MS spectra with literature data [49]. Five isomers of diarylated cyclohexenes were found in the GC, and their composition was confirmed by MS (m/z = 234, M^+^). 

## 4. Conclusions

The Heck coupling of iodobenzene with cyclohexene performed with Pd(OAc)_2_ and PdCl_2_(PhCN)_2_ as catalyst precursors in molten [Bu_4_N]Br salt resulted in a maximum yield of monoarylated products amounting to *ca.* 60%. The soluble palladium species, [Bu_4_N]_2_[PdX_4_] complexes, and Pd(0) nanoparticles stabilized by tetrabutylammonium salt were most probably responsible for the observed reactivity. The same precursors used in DMF presented a very low catalytic activity. In contrast, palladium catalysts containing Pd(II) supported on Al_2_O_3_ and alumina-based mixed oxides, Al_2_O_3_-ZrO_2_, Al_2_O_3_-CeO_2_, Al_2_O_3_-Fe_2_O_3_, were quite effective in DMF, producing up to 46% of monoarylated products, with 4-phenylcyclohexene (**3**) being the predominant one. The same catalysts used in [Bu_4_N]Br presented a lower selectivity and produced 3-phenylcyclohexene (**2**) and 4-phenyl-cyclohexene (**3**) in comparable amounts, with a slight excess of **2**. Different results obtained in with homogeneous and heterogeneous precursors can be explained by the contribution in the reaction course of insoluble, supported palladium species besides of soluble ones. The most attractive and promising results, up to 81% of monoarylated products, were obtained with heterogenized palladium catalysts in DMF, with KOH as the base. Excellent selectivity to **3** was also noted under such conditions. 
